# The Genome Analysis of *Methylobacterium populi* YC-XJ1 with Diverse Xenobiotics Biodegrading Capacity and Degradation Characteristics of Related Hydrolase

**DOI:** 10.3390/ijms21124436

**Published:** 2020-06-22

**Authors:** Xianjun Li, Junhuan Wang, Yang Jia, Aikebaier Reheman, Yanchun Yan

**Affiliations:** 1 Graduate School, Chinese Academy of Agricultural Sciences, Beijing 100081, China; lixianjun1978@126.com (X.L.); wangjunhuan@caas.cn (J.W.); jia_yang@outlook.com (Y.J.); 2 Key Laboratory of Toxicology, Ningde Normal University, Ningde 352100, China

**Keywords:** *Methylobacterium populi*, genome sequencing, hydrolase, quizalofop-p-ethyl, diethyl phthalate, degradation

## Abstract

*Methylobacterium populi* YC-XJ1 isolated from desert soil exhibited a diverse degrading ability towards aromatic oxyphenoxypropionic acid esters (AOPPs) herbicide, phthalate esters (PAEs), organophosphorus flame retardants (OPFRs), chlorpyrifos and phoxim. The genome of YC-XJ1 was sequenced and analyzed systematically. YC-XJ1 contained a large number of exogenous compounds degradation pathways and hydrolase resources. The quizalofop-p-ethyl (QPE) degrading gene *qpeh2* and diethyl phthalate (DEP) degrading gene *deph1* were cloned and expressed. The characteristics of corresponding hydrolases were investigated. The specific activity of recombinant QPEH2 was 0.1 ± 0.02 U mg^−1^ for QPE with *k*_cat_/*K*_m_ values of 1.8 ± 0.016 (mM^−1^·s^−1^). The specific activity of recombinant DEPH1 was 0.1 ± 0.02 U mg^−1^ for DEP with *k*_cat_/*K*_m_ values of 0.8 ± 0.02 (mM^−^^1^·s^−^^1^). This work systematically illuminated the metabolic versatility of strain YC-XJ1 via the combination of genomics analysis and laboratory experiments. These results suggested that strain YC-XJ1 with diverse xenobiotics biodegrading capacity was a promising candidate for the bioremediation of polluted sites.

## 1. Introduction

Quizalofop-p-ethyl (QPE), a unitary R configuration aromatic oxyphenoxypropionic acid esters (AOPPs) herbicide, was predicted to rapidly increase in coming years in an effort to overcome the widespread appearance of glyphosate-resistant weeds [1]. Due to the widespread use of QPE, some detrimental effects had been reported, such as: causing liver injury of human [2]; causing alterations of gene expression in fatty acid degradation pathways of the HepaRG cell line [1]; endocrine-disrupting and acute toxicity to zebrafish [3]; phytotoxicity to *Lemna minor* [4]; toxicity to soil microorganisms [5]. According to the World Health Organization (WHO) definitions of adverse drug reactions and U.S. Environmental Protection Agency (EPA) toxicity class III, QPE was toxic chemicals, already prohibited by the European Union.

Phthalate esters (PAEs) are one of the most frequently detected persistent organic pollutants in the environment [6]. Diethyl phthalate (DEP) belongs to the family of PAEs, which are the most commonly used plasticizer in China [7]. An investigation on particulate and gas-phase distribution of PAEs in Nanjing (China), approximately 75 to 89.2% of phthalate esters were present in the atmosphere in vapor form, DEP is 3.4 ng m^−3^ [8]. An analysis of gas-particle partitioning shown the vapor phase distribution of DEP in the atmosphere of Paris in France is 93.8% [9]. DEP concentration is 406 pg cm^−3^ in gas-phase in the arctic which is the largest concentration of among 6 tested PAEs [10]. Due to the widespread use of DEP in personal care products, plastics and medical devices, some detrimental effects had been reported, such as chronic toxicity in male wistar rats [11]; hepatotoxicity to 21-day-old male and female weanling pups of Wistar rats [12]; causing changes of certain liver and muscle enzyme activity to freshwater fish *Cirrhina mrigala* [13]; causing severe impairment of lipid metabolism coupled with toxic injury of the liver in young male Sprague-Dawley rats [14]; causing neurotoxicity in zebrafish embryos [15].

Triphenyl phosphate (TPhP), as an aryl organophosphorus flame retardants (aryl-OPFRs), was extensively used in a variety of industries, such as plastics, furniture, textile, electronics, construction, vehicle and petroleum [16]. Due to its chemical properties, OPFRs were not chemically bonded to the final products, which resulted in an easy release to the environment [17], such as indoor dust [18], drinking water [19], rivers [20]. Some toxicological effects of TPhP had been reported, such as reproductive and developmental toxicity of zebrafish [21], inducing oxidative stress and endocrine disruption in mice [22].

Remediation of polluted sites using a microbial process has been proven effective and reliable due to its eco-friendly features [23]. Therefore, a series of microorganisms with degradation capacity have been isolated in recent years. Some strains from *Pseudomonas* sp. [24], *Aquamicrobium* sp. [25], *Acinetobacter* sp. [26] and *Rhodococcus* sp. [27] were identified to degrade QPE. Some strains from *Acinetobacter* sp. [28], *Sphingomonas* sp. [29], *Paenibacillus* sp. [30] and *Ochrobactrum* sp. [31] were identified to degrade DEP. Some strains from *Brevibacillus brevis* [32], *Sphingobium* sp., *Sphingomonas* sp. [33], *Roseobacter* sp. [34], *Rhodococcus* sp. and *Sphingopyxis* sp. [35] were identified to degrade TPhP. However, most of these isolates have a single function and can only degrade one kind of organic pollutants. In this research, we report the *Methylobacterium populi* YC-XJ1 with diverse degrading function isolated from desert soil. The whole genome was sequenced and analyzed systematically. Characteristics of related hydrolase were further investigated.

## 2. Results

### 2.1. Substrates Utilization Test

**Figure 1:**
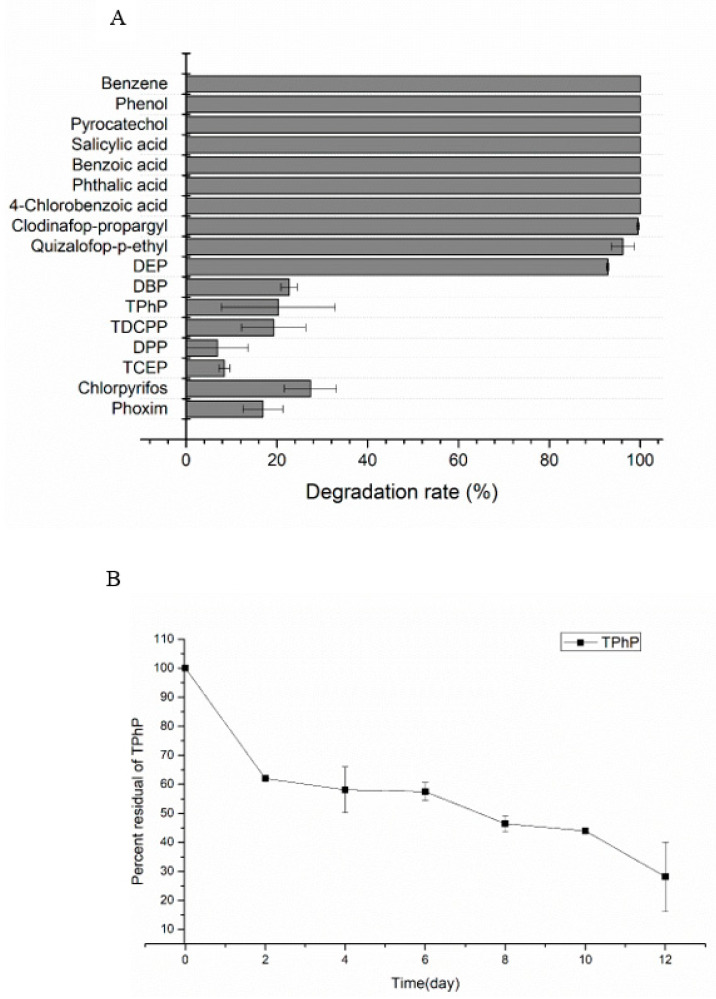
The degradation of various substrates by *Methylobacterium populi* YC-XJ1. (**A**) Substrate specificity of YC-XJ1; (**B**) Degradation of TPhP by YC-XJ1).

One After 72 h incubation, aromatic compounds including benzene, phenol, pyrocatechol, salicylic acid, benzoic acid, phthalic acid, 4-chlorobenzoic acid and haloxyfop-p-methyl, diclofop-methyl, fluazifop-p-butyl were completely degraded (100%), as shown in Figure 1A. The AOPPs including quizalofop-p-ethyl and clodinafop-propargyl were degraded by 96.2%, 99.5% respectively. The PAEs including dibutyl phthalate (DBP) and DEP were degraded by 93% and 23% respectively. The degradation rate of TPhP, diphenyl phosphate (DPP), tris(1,3-dichloro-2-propyl) phosphate (TDCPP) and tris(2-chloroethyl) phosphate (TCEP) were 20.3%, 6.9%, 19.3% and 8.4% respectively. The compounds with phosphorous ester bond including chlorpyrifos and phoxim were degraded by 27.3% and 16.9% respectively.

The characteristics on degrading TPhP by strain YC-XJ1 were further explored; the degradation ratio of TPhP in initial concentration 50 mg/L was 72% within 12 days in Figure 1B, the optional pH of TPhP by strain YC-XJ1 was pH8 (Figure S1).

### 2.2. Genome Properties

**Figure 2:**
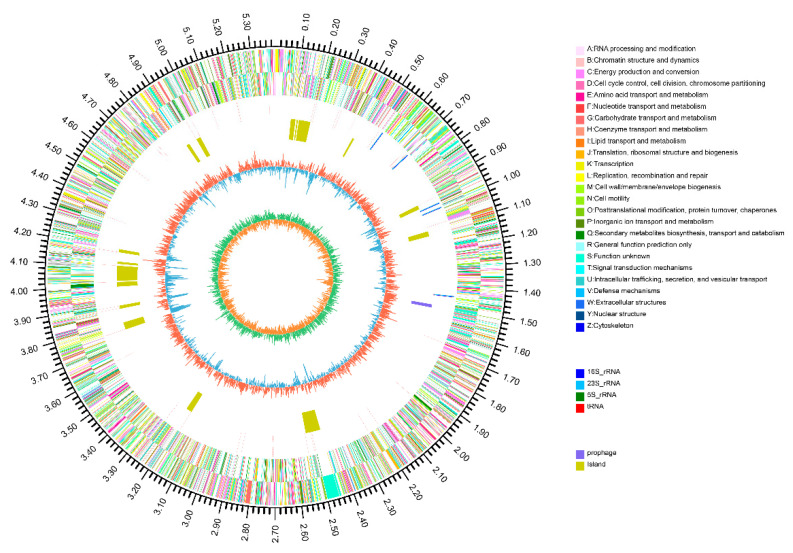
Schematic of the complete *Methylobacterium populi* YC-XJ1 genome. Circles are numbered from the outermost (first) to the innermost (eighths) circle and include the following features: the scale line, with each major tick representing 0.1 Mb (first circle); coding DNA sequences on forward and reverse chains, with different colors based on clusters of orthologous groups of proteins (COGs) categories (second and third circles); rRNA and tRNA (fourth circle); prophage (fifth circle); gene island (sixth circle); GC content (seventh circle); GC skew (eighth circle). The circular representation was drawn using Circos 0.64.

The *Methylobacterium populi* YC-XJ1 genome was assembled into one scaffold of 5,395,646 bp with a G + C content of 69.36%. Gene prediction indicates a total gene length of 4,685,898 bp, with a GC content of 69.95% in the gene region and 65.47% in intergenetic region. The gene length/genome ratio is 86.85%. The genome contains 10 rRNA genes, 58 tRNA genes, 1 prophage (16,754 bp); 19 gene island (549,538 bp) and 5375 coding sequences (CDSs) (Figure 2). 4387 (81.6%) CDSs have assigned predicted functions (Figure 2). Gene numbers were multiply annotated in databases as follows: non-redundant (NR) (3547), Swiss-Prot (3174), Cluster of Orthologous Groups of proteins (COGs) (4170), Kyoto Encyclopedia of Genes and Genomes (KEGGs) (2310), Gene Ontology (GO) (3458), Pfam (3853), Carbohydrate-Active enzymes (CAZy) (166), Pathogen Host Interactions (PHI) (659), Virulence Factors Database (VFDB) (435) and Comprehensive Antibiotic Research Database (CARD) (249), as shown in Table S1.

### 2.3. ANI Analysis

To further confirm the identification of YC-XJ1, average nucleotide identity (ANI) analysis was performed using whole-genome data between YC-XJ1 and other closely related 7 genome sequence of *Methylorubrum* strains which retrieved from the GenBank database. 

A heatmap was constructed based on ANI values (Figure S2), the whole genome phylogenetic tree showed YC-XJ1 was closestly relative to *Methylobacterium populi* BJ001, and 97.9% identical to the genome of *Methylobacterium populi* BJ001, with 88.6% coverage of the genome.

### 2.4. Collinearity Analysis

The collinearity analysis of the YC-XJ1 genome and the closest BJ001 genome was conducted. The collinearity and non collinearity regions of the genome of strain YC-XJ1 and BJ001 were shown in Figure S3. There was not a large area of gene location transfer and gene rearrangement between YC-XJ1 and BJ001 genome.

### 2.5. Gene Function Analysis

#### 2.5.1. COG Categories Reveal Metabolism-Related Functions

Since 4170 sequences were annotated to 4 COG categories and 21 COGs types. 1342 genes (32.2%) were annotated to the metabolism of COG categories and the metabolism COG categories included 8 COG types (Figure S4). To determine the degrading potential of *Methylobacterium populi* YC-XJ1, specific COGinvolved in xenobiotics metabolism were analyzed. Among the metabolism, the four most abundant COG types were the energy production and conversion (270 genes, 6.5%), the amino acid transport and metabolism (265 genes, 6.4%), the inorganic ion transport and metabolism (248 genes, 5.9%), the carbohydrate transport and metabolism (175 genes, 4.2%).

With respect to the carbohydrate transport and metabolism, It concluded a series of COG0477 (major facilitator Superfamily), COG1472 (hydrolase family 3), COG2273 (hydrolase family 16), COG1554 (hydrolase family 65), COG0647 (hydrolase), COG4993 (dehydrogenase) and COG2133 (dehydrogenase) involved the degradation of xenobiotics.

COG0477 catalyzes the transport of various substrates, including carbohydrates, ions, and other small molecules. It was referred to as a secondary active transporter [36]. COG1472 (hydrolase family 3) was a beta-glucosidase or beta-hexosaminidase. COG2273 (hydrolase family 16) was a glycoside hydrolase. COG0647 (hydrolase) was a haloacid dehalogenase. COG1554 (hydrolase family 65) was a kojibiose phosphorylase. COG4993 (dehydrogenase) was a methanol dehydrogenase. COG2133 (dehydrogenase) was a glucose or sorbosone dehydrogenase. The wide diversity of gene function reveals a high potential for *Methylobacterium populi* YC-XJ1 in carbohydrate degradation.

Another obvious characteristic of *Methylobacterium populi* YC-XJ1 was that 1367 genes (32.8%) were annotated to Function unknown, implying many proteins with degrading function have not yet to be identified (Figure S4).

#### 2.5.2. GO Terms Reveal Biological Relevance

To determine the biological relevance of the *Methylobacterium populi* YC-XJ1 gene pool, genes were categorized by GO analysis based on matches to sequences of known functions in three categories: biological process, cellular component, and molecular function (Figure S5). GO terms of the molecular function (2733 genes) were the most abundant, followed by biological process (2473 genes) and cellular component (1674 genes).

For *Methylobacterium populi* YC-XJ1 isolated from the desert soil under higher temperature alkaline environment, genes in molecular function category fell into 12 sub-functions, with most involved in catalytic activity (GO:0003824, 2023, 48.0%) and binding (GO:0005488, 1385, 32.9%); genes in the biological process category fell into sixteen sub-functions, with a large proportion of genes being involved in metabolic process (GO:0008152, 1910, 27.0%), cellular process (GO:0009987, 1558, 22.0%), and single-organism process (GO:0044699, 1354, 19.1%); genes in the cellular component category fell into 12 sub-functions, with most involved in functions of membrane (GO:0016020, 1092, 26.2%), membrane part (GO:0044425,992, 23.8%), cell (GO:0005623, 890, 21.4%), and cell part (GO:0044464, 870, 20.9%).

Based on the GO annotation of *Methylobacterium populi* YC-XJ1, genes in the catalytic activity, metabolic process, and cellular process sub-functions were the three most abundant.

#### 2.5.3. CAZy Families Reveal Carbohydrate-Active Enzyme Genes

To further understand the carbohydrate degradation capacity of YC-XJ1, genes were annotated against the CAZy database. YC-XJ1 contained 166 gene counts distributed unequally among glycoside hydrolases (GHs; 26.51%), carbohydrate esterases (CEs; 16.87%), glycosyl transferases (GTs; 46.39%), auxiliary activities (AAs; 6.02%), and carbohydrate binding modules (CBMs; 1.20%) (Figure S6). With the highest gene counts percent, the enzymes of the GHs, CEs and GTs families played key roles in the cleavage of substrates [37].

Carbohydrate esterases (CEs) catalyzed the de-O or de-N-acylation by removing the ester decorations from carbohydrates [38]. CEs were currently classified into 15 families in the CAZy database, and CEs had important significance as biocatalysts in a variety of bioindustrial processes and applications [39]. Among carbohydrate esterases in YC-XJ1, there were CE10 family (9 genes) associated with the degradation of AOPPs, apart from CE1 family (6 genes). CE10 family included carboxyl esterase (EC 3.1.1.3); arylesterase (EC 3.1.1.2); acetylcholinesterase (EC 3.1.1.7); cholinesterase (EC 3.1.1.8); sterol esterase (EC 3.1.1.13). CE1 family include carboxylesterase (EC 3.1.1.1); cinnamoyl esterase (EC 3.1.1.73); acetyl xylan esterase (EC 3.1.1.72); feruloyl esterase (EC 3.1.1.73).

The glycoside hydrolases (GH) was a well-known group of enzymes that hydrolyze the glycosidic bond of carbohydrates. GH members in YC-XJ1 included glycoside hydrolase family 15 (GH15), oxidoreductase (GH109).

#### 2.5.4. Analysis of Relative Metabolic Pathways

To explore the critical metabolic pathways involved in degradation of xenobiotics by YC-XJ1, 2310 (43.0%) genes were involved in 223 metabolic pathways in KEGG database. The summary of the annotated metabolic pathways was shown in Figure S7. Most genes involved in “metabolism”, gene number 1438 and gene percent 62.3%, more than any other categories including cellular processes, human diseases, organismal systems, genetic information processing and environmental information processing. Among metabolism, “energy metabolism” and “carbohydrate metabolism” were two primary functions of YC-XJ1 in KEGG annotations (Figure S8).

“Energy metabolism” (234, 10.1%) represented “the plant of power” in bacterium. Methane metabolism (ko00680, 56 genes) and oxidative phosphorylation (ko00190,53) in YC-XJ1 indicated that YC-XJ1 could utilize C1 compound efficiently and released chemical energy ATP which plays a key role in carbohydrate metabolism. Carbon fixation pathways in prokaryotes (ko00720, 35) and carbon fixation in photosynthetic organisms (ko00710, 18) implied the super capacity of carbon fixation.

“Carbohydrate metabolism” (346, 15.0%) contained more gene number than “Energy metabolism” (234, 10.1%). The largest gene proportion of “Carbohydrate metabolism” participated in glyoxylate and dicarboxylate metabolism (ko00630, 66).

The most important KEGG second category was “xenobiotic biodegradation and metabolism” (96, 4.2%), as shown in Table 1. Degradation pathways of single benzene ring aromatic compounds such as benzoate, styrene, toluene and xylene were annotated, and that was corresponding with the result of previous experiments. Aminobenzoate and nitrotoluene degradation pathway also were annotated, implying the strain YC-XJ1 has the function of deamination and denitrifying. Dioxin, chlorocyclohexane, chlorobenzene and fluorobenzoate halogenated aromatic compounds, atrazine and polycyclic aromatic hydrocarbon degradation pathway were annotated, explaining the dehalogenation function and the broad substrate of YC-XJ1.

### 2.6. Characteristics of QPEH2 Hydrolase

**Figure 3:**
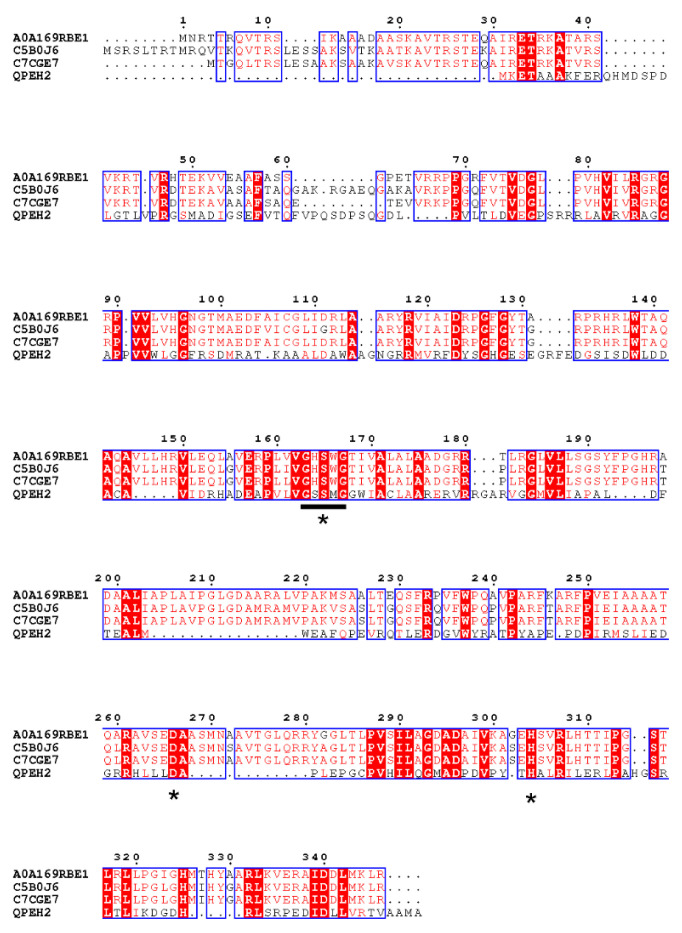
The sequence alignment of QPEH2 with the most closely related proteins. (A0A169RBE1, α/β hydrolase of *Methylorubrum populi*, available in UniProt Knowledgebase; C5B0J6, Putative hydrolase of *Methylorubrum extorquens* ATCC 14718; C7CGE7, Putative hydrolase of *Methylorubrum extorquens* DSM 6343, available in the UniProt Knowledgebase. The conserved hydrolase motif (G-X-S-X-G) was underlined, and the amino acids that form the catalytic triad (Ser-Asp-His) was indicated by asterisks. The identical amino acid residues were shown in red color).

One gene was selected according to the annotation results, and named as *qpeh2*. The cloned *qpeh2* gene was 1104 bp in length with a GC content of 72.7% and encoded a protein of 367 amino acids with a calculated molecular mass of 39,840 Da. No signal peptide in protein sequence. The alignment analysis results showed QPEH2 contained the conserved esterase family sequence motif (G-X-S-X-G) and the catalytic triad (Ser-Asp-His) in Figure 3. This suggested that QPEH2 was a member of esterase family.

**Figure 4:**
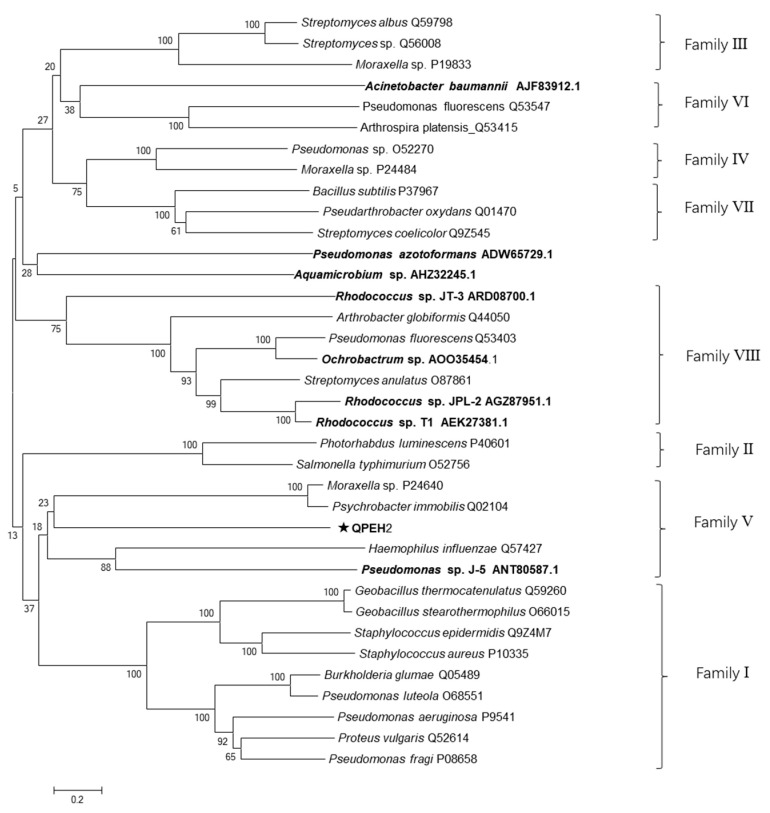
The phylogenetic analysis of QPEH2. The phylogenetic tree was constructed using Mega 5.0 by the neighbor-joining method, bootstrapping of 1000 replicates and Poisson model, and the details of sequences were showed in Table S2. The bold-type letters represent all the QPE-degrading esterases reported.

Members of subfamilies I–VIII and all esterases of QPE-degrading reported were used to construct a phylogenetic tree to verify the evolutionary relationship between QPEH2 and other esterases. Most of the 8 esterases of QPE-degrading reported were included in family V and VIII (Figure 4). The QPEH2 belonged to family V. The QPEH2 shared 25.7% identity with QpeH which is the most closely related QPE-degrading hydrolase from *Pseudomonas* sp. J-5. This result revealed significant differences of QPEH2 with other QPE-degrading hydrolases.

**Figure 5:**
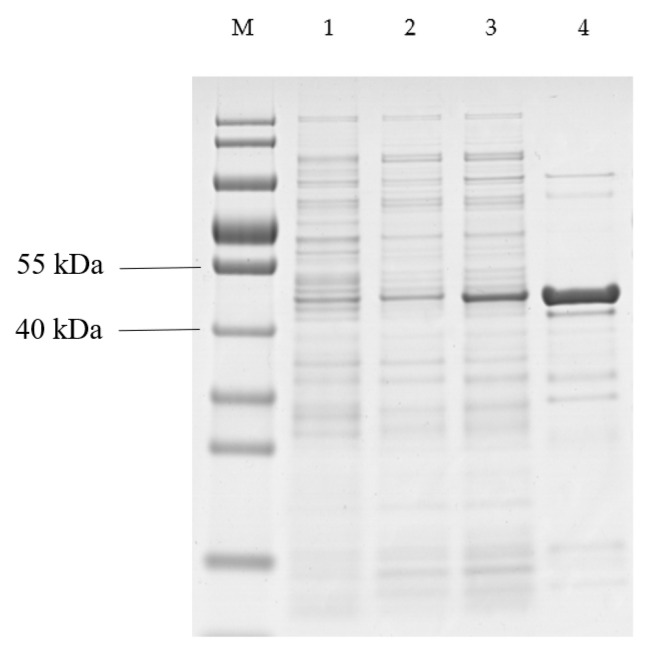
SDS-PAGE analysis of QPEH2 purification. Lane M: protein marker; lane 1: total protein of *E. coli* (pET32a); lane 2: total protein of *E. coli* (pET32a-*qpeh2*) without induction; lane 3: total protein of *E. coli* (pET32a-*qpeh2*) after induction with IPTG; lane 4: purified recombinant QPEH2.

Purification was analyzed by SDS-PAGE and the result showed that an approximate 45 kDa protein was obtained. The recombinant QPEH2 most likely corresponded to the 5 kDa of His·Tag/S·Tag sequences fused to the expected 40 kDa *qpeh2* gene product (Figure 5).

**Figure 6:**
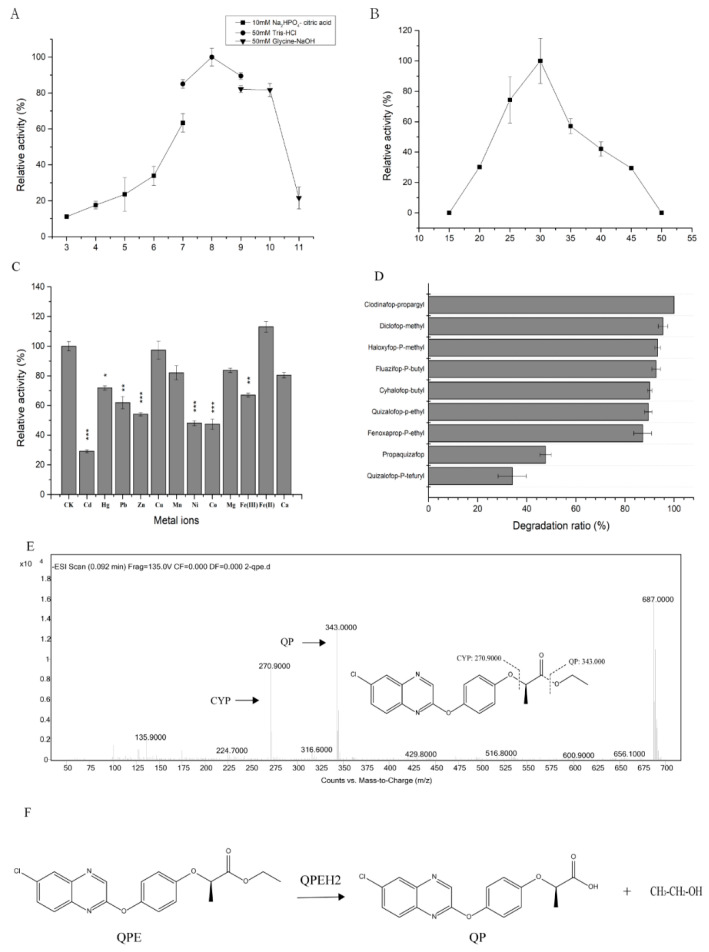
Characteristics of the hydrolase QPEH2. (**A**) pH; (**B**) temperature; (**C**) metal ions; (**D**) substrate specificity of QPEH2; (**E**) the HPLC-MS analysis of intermediates of QPE degradation by QPEH2; (**F**) the metabolic pathway of QPE by QPEH2; the asterisks (***) represent significant differences (*p* ≤ 0.001); the asterisks (**) represent significant differences (*p* ≤ 0.01); the asterisks (*) represent significant differences (*p* ≤ 0.05).

The recombinant QPEH2 exhibited high levels of activity at pH7-10, with an optimum pH of 8.0 (Figure 6A). The enzyme was active at 20–45 °C, with an optimum temperature of 30 °C (Figure 6B).

Most of the metal ions decreased enzymatic activity of QPEH2, especially, the Cd^2+^, Pb^2+^, Zn^2+^, Ni^2+^, Co^2+^, Fe^3+^ has a significant inhibitory effect (Figure 6C), only Fe^2+^ increased enzymatic activity at a concentration of 1 mM.

The substrate specificity of QPEH2 was assessed with various AOPPs (Figure 6D). The catalytic efficiency of QPEH2 toward different AOPPs herbicides in descending order was as follows: clodinafop-propargyl > diclofop-methyl > haloxyfop-p-methyl > fluazifop-p-butyl > cyhalofop-butyl > fenoxaprop-p-ethyl > QPE > propaquizafop > quizalofop-p-tefuryl.

The specific activity of recombinant QPEH2 was 0.1 ± 0.02 (U mg^−1^) for QPE with *k*_cat_/*K*_m_ and *V*_max_ value of 1.8 ± 0.016 (mM^−1^·s^−1^) and 0.38 ± 0.14 (µM·s^−1^), *K*_m_ of 83.87 ± 30.34 µM.

In addition, the metabolites of QPE were identified by HPLC-MS analysis. Two metabolites of quizalofop-p (QP) and (4-(6-Chloroquinoxalin-2-yloxy) phenol (CYP) were detected (Figure 6E). Proposed pathway of QPE hydrolysis by QPEH2 (Figure 6F).

### 2.7. Enzymatic Characteristics of DEPH1

One gene was selected according to the annotation results, and named as *deph1.* The cloned *deph1* gene was 843 bp in length with a GC content of 72.7% and encoded a protein of 280 amino acids with a calculated molecular mass of 29,714 Da. No signal peptide in protein sequence. The alignment analysis results showed DEPH1 contained the conserved esterase family sequence motif (G-X-S-X-G) and the catalytic triad (Ser-Asp-His) in Figure S9. This suggested that DEPH1 was a member of esterase family. Members of subfamilies I–VIII and DEPH1 were used to construct a phylogenetic tree to verify the evolutionary relationship between DEPH1 and other esterases. The DEPH1 belonged to Family VII (Figure S10). The DEPH1 shared 28.6% identity with P37967 which is the most closely related carboxylic ester hydrolase from *Bacillus subtilis*. This result revealed significant differences of DEPH1 with other hydrolases reported.

Purification was analyzed by SDS-PAGE and the result showed that an approximate 48 kDa protein was obtained. The recombinant DEPH1 most likely corresponded to the 18 kDa of Trx·Tag/His·Tag/S·Tag sequences fused to the expected 30 kDa *deph1* gene product (Figure S11).

**Figure 7:**
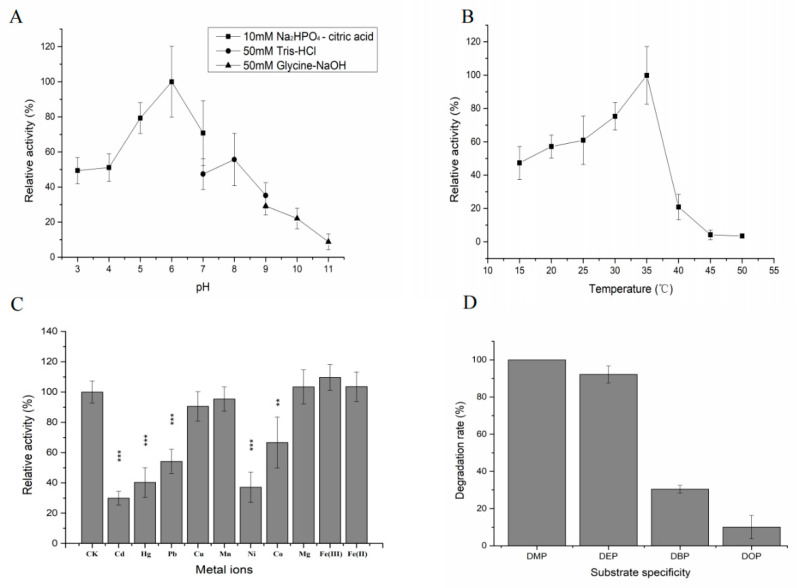
Characteristics of the hydrolase DEPH1. (**A**) pH; (**B**) temperature; (**C**) metal ions; (**D**) substrate specificity of DEPH1; the asterisks (***) represent significant differences (*p* ≤ 0.001); the asterisks (**) represent significant differences (*p* ≤ 0.01).

The recombinant DEPH1 exhibited high levels of activity toward DEP at pH 4–8, with an optimum pH 6.0 (Figure 7A). The enzyme was active at 20–35 °C, with an optimum temperature of 35 °C (Figure 7B).

Most of the metal ions decreased enzymatic activity of DEPH1 toward DEP, such as Cd^2+^, Pb^2+^, Zn^2+^, Ni^2+^, Co^2+^ and Mn^2+^. Only Mg^2+^ and Fe^3+^ increased enzymatic activity (Figure 7C).

The substrate specificity of DEPH1 was assessed with 4 PAEs (Figure 7D). The catalytic efficiency of DEPH1 toward different PAEs in descending order was as follows: DMP > DEP > DBP > DOP (Figure 7D).

The specific activity of recombinant DEPH1 was 0.1 ± 0.04 (U mg^−1^) for DEP with *k*_cat_/*K*_m_ and *V*_max_ value of 0.8 ± 0.02 (mM^−1^·s^−1^) and 0.23 ± 0.05 (µM·s^−1^), *K*_m_ of 1.04 ± 0.27 mM.

## 3. Discussion

According to the results of substrates utilization test, YC-XJ1 showed the ability to degrade a variety of different types of organic pollutants, such as QPE (AOPPs herbicide), DEP (PAEs), TPhP (OPFRs) chlorpyrifos and phoxim (organophosphate insecticides). Based on the reports, *Acinetobacter* sp. DL-2 could degrade 90.1% of QPE (50 mg/L) within 120 h [26], *Aquamicrobium* sp. FPB-1 could degrade 98.5% of QPE (100 mg/L) within 40 h [25]. Compared to them, YC-XJ1could degrade 96.2% of QPE (50 mg/L) within 72 h. *Pseudomonas* sp. DNE-S1 could degrade 90% of DEP (153 mg/L) within 60 h [29], *Acinetobacter* sp. M673 could degrade 80% of DEP (0.1 mM/L) within 12 h [28]. Compared to them, YC-XJ1 could degrade 93% of DEP (50 mg/L) within 72 h. Few TPhP degrading bacteria have been reported. *Brevibacillus brevis* could degrade 92.1% of TPhP (3 μmol/L) within 5 days [32], *Rhodococcus* sp. YC-JH2 could degrade 37.36% of TPhP (50 mg/L) within 7 days, *Sphingopyxis* sp. YC-JH3 could efficiently degrade 96.2% of TPhP (50 mg/L) within 7 days [35]. In my study, YC-XJ1 could degrade 72% of TPhP (50 mg/L) within 12 days.

The ANI is one of the most robust measurements of genomic relatedness between bacterial strains, and has a great potential in the taxonomy classification of bacteria as a substitute for the traditional technique. The proposed and generally accepted species boundary for ANI values are 95–96% [40]. The strain YC-XJ1 was identified as *Methylobacterium populi* finally.

Due to the great phenotypic plasticity, the Members of the *Methylobacterium* genus occupy different habitats and distributed in the natural environment widely [41]. They potentially play an important role in mitigating ozone depletion resulting from methyl chloride and methyl bromide emissions [42]. According to the reports, *Methylobacterium* genus could degrade polycyclic aromatic hydrocarbon [43], 2,2-bis(p-chlorophenyl)-1,1-dichloroethylene [44], 2,4,6-trinitrotoluene, 4-Nitro-2,4-diazabutanal [45], isoprene [46] and other compounds. In this study, it was reported for the first time that *Methylobacterium* could degrade AOPPs herbicide and OPFRs and PAEs. Aromatic carboxylic acid ester bond and organic phosphoester bond were preferred by strain YC-XJ1.

For atrazine degradation pathway annotated, there was a large percent (10 genes) participate in it. Although atrazine was not detected by previous substrate experiment, it brings up another hint that atrazine, a herbicide different from AOPPs on molecular structure, may be an important substrate of YC-XJ1.

The overall gene functions displayed that the strain YC-XJ1 was a promising candidate for organic degradation. Based on microbial function analysis, the future research would develop an efficient modified engineered bacterium for application.

Environmental factors such as pH and temperature can affect enzyme activity and stability [47]. The optimal condition of QPEH2 was consistent with the reported characteristics of esterase QpeH [24]. Metal ions also played a key role in the activity of enzymes. Only Fe^2+^ could serve as activator for QPEH2, different from the previous report that 1 mM Ca^2+^ and Cd^2+^ strongly stimulated enzyme activity of QpeH from *Pseudomonas* sp. J-2 [24].

Among all substrates, quizalofop-p-tefuryl degradation rate was the lowest, and it was also the only AOPPs with tetrahydrofuran ring structure in the side chain of carboxylate. It was speculated that complex side chain and steric hindrance existed to affect the activity of QPEH2.

For the other reported QPE hydrolase, the specific activities of FeH from *Rhodococcus ruber* JPL-2 were 1.16 (U mg^−1^) for QPE with *k*_cat_/*K*_m_ value of 3.25 mM^−1^·s^−1^ [48]; 4.12 ± 0.25 (U mg^−1^) of ChbH from *Pseudomonas azotoformans* QDZ-1, with *k*_cat_/*K*_m_ value of 6.57 µM^−1^·s^−1^ [49]; 7.95 ± 0.22 (U mg^−1^) of FpbH from *Aquamicrobium* sp. FPB-1, with *k*_cat_/*K*_m_ value of 12.85 µM^−1^·s^−1^ [25]. QPEH2 did not show superior degradation efficiency comparing to them.

According to the identification of metabolites, QPE was converted to QA and ethanol, consistent with the result of QpeH reported [24].

The optimal condition (pH 6.0 and 35 °C) of DEPH1 was different from reported BaCEs04 with optimal condition (pH 7.5 and 60 °C). Mg^2+^ and Fe^2+^ could serve as activator for DEPH1, consistent with the characteristics of BaCEs04 [50].

Among all substrates of DEPH1, the degradation rate of DOP was only 10.03%. It was obvious that the enzyme activity decreased with the extension of the side chain of carboxylate.

For the reported dialkyl PAEs hydrolase, the specific activities of BaCEs04 from *Bacillus velezensis* SYBC H47 towards DEP was 21.27 ± 1.18 (U mg^−1^) [50]; The *k*_cat_/*K*_m_ and *K*_m_ of dialkyl PAEs hydrolase from *Camelimonas* sp. for DEP was 9.8234 ± 0.013 (mM^−1^·s^−1^) and 1.5289 ± 0.021 (mM) [51]; The *k*_cat_/*K*_m_ and *K*_m_ of dialkyl PAEs hydrolase from *Acinetobacter* sp. M673 for DEP was 7.34 ± 0.34 (μM^−1^·s^−1^) and 1.943 ± 0.012 (µM) [28]. DEPH1 did not show superior degradation efficiency comparing to them.

Although QPEH2 and DEPH1 did not show superior degradation efficiency, more than 40 similar hydrolases genes were screened based on genome annotation, and *qpeh2* and *deph1* were only two of them whose activity was verified. The strong degradation activity of YC-XJ1 on the substrate was probably the result of the interaction of multiple hydrolases in vivo, or QPEH2 and DEPH1 were not the hydrolases with the fastest degradation rate. Although a large number of gene functional verification experiments have been carried out, hydrolases of TPhP were still unknow. In order to elaborate the degradation mechanism of TPhP, It was necessary to find the corresponding hydrolase.

Bacteria with the ability to degrade multiple types of organic pollutants have rarely been reported. Methylobacter populi was often isolated in extremely polluted areas and has tolerance to extreme environments. In future, the research would focus on the use of transgenic engineering to transfer QPE and DEP degradation genes from other species into the genome of YC-XJ1 to improve the degradation ability. It was hoped that the *Methylobacterium populi* YC-XJ1 could be modified as engineered bacteria for practical application in bioremediation of polluted sites.

## 4. Materials and Methods

### 4.1. Chemicals and Reagents

Standards of clodinafop-propargyl, diclofop-methyl, haloxyfop-p-methyl, fluazifop-p-butyl, cyhalofop-butyl, fenoxaprop-p-ethyl, propaquizafop, quizalofop-p-tefuryl, QPE (98.7% of purity) were purchased from Shenyang Research Institute of Chemical Industry Co., Ltd. (Shenyang, China). QP (>95% of purity), CYP (95% of purity), and quinoxaline (95% of purity) were purchased from Sigma-Aldrich (St. Louis, MO, USA). Stock solutions (1 × 10^4^ mg/L) of all standard substance were prepared by dissolving in methanol (HPLC grade).

Standards of DMP, DBP, MEP, DEP, DOP, TPhP, DPP, TDCPP, TCEP, chlorpyrifos, phoxim, benzene, 4-chlorobenzoic acid, phenol, pyrocatechol, salicylic acid, benzoic acid, phthalic acid were also made by dissolving in methanol (1 × 10^4^ mg/L).

### 4.2. Media

Mineral salts medium (MSM) composed of 1.5 g NH_4_NO_3_, 0.5 g KH_2_PO_4_·12H_2_O_,_ 1.5 g K_2_HPO_4,_ 0.2 g MgSO_4_·7H_2_O, 0.5 g NaCl and 1‰ (v/v) trace element solution (TES) in 1.0 L water. Trace element solution (TES) contained FeSO_4_·7H_2_O (2.0 g/L), ZnSO_4_ (0.1 g/L), CuSO_4_·5H_2_O (0.03 g/L), MnCl·4H_2_O (0.03 g/L), CoCl·7H_2_O (0.3 g/L), Na_2_MoO_4_·2H_2_O (0.03 g/L), Na_2_WO_4_·2H_2_O (0.02 g/L). NaCl solution (200 mg/mL) contained 20 g NaCl and 100 mL PBS (10 mM) buffer. The pH of all media was adjusted to 7.0 ± 0.2, then all media were sterilized at 121 °C for 20 min.

### 4.3. Analytical Methods

Three biological replications of 10 mL aqueous samples were extracted with the equal volume of dichloromethane, and 800 µL extracts were evaporated in fuming cupboard. The residues were dissolved in 800 µL methanol and the solution was filtered through 0.22 µm membrane (ANPEL, Shanghai, China) before determined by high performance liquid chromatography (HPLC) system (1200 series, Agilent Technologies Inc., Santa Clara, California, USA) equipped with a C18 column (Agilent Eclipse XDB, 5 µm, 4.6 × 150 nm) and a diode array detector (DAD), see Table S3 for details of the settings.

Three biological replications of 10 mL aqueous samples were extracted with the equal volume of dichloromethane, and 800 µL extracts were evaporated in fuming cupboard. The residues were dissolved in 800 µL acetonitrile and the solution was filtered through 0.22 µm membrane before detected by gas chromatograph system (GC-2010, SHIMADZU, Kyoto, Japan) equipped with a HP-5 capillary column (inradium—0.25 mm, length—30 m, membrane thickness—0.25 µm) and a flame ionization detector (FID) [35], see Table S3 for details of the settings.

### 4.4. Substrates Utilization Tests

An inoculum of strain YC-XJ1 was prepared and used to determine the biodegradability of different compounds. 50 mg/L substrate was set as the initial concentration, i.e., 10 mL MSM (pH 8.0) containing 50 µL stock solutions and 1% *Methylobacterium populi* YC-XJ1 bacterial suspension (OD_600_ = 0.7), was incubated at 35 °C and 180 rpm for 3 days. Each treatment was performed in three replicates, and the samples without inoculation were set as a control. The residual concentration was detected by HPLC or GC as described above (Section 4.3), and standard curves of all substrates were prepared previously, see Figure S12 for details.

### 4.5. Genomic DNA Extractions, Library Construction and Sequencing

The method was used as described in [52].

### 4.6. Gene Prediction and Annotation

The data generated from PacBio and Illumina platform were used for bioinformatics analysis. All of the analyses were performed using I-Sanger Cloud Platform (www.i-sanger.com) from Majorbio Bio-pharm Technology Co., Ltd. (Shanghai, China). The coding sequence (CDS) was predicted with Glimmer v3.02. The tRNA and rRNA were predicted with tRNA-scan-SE v2.0 and Barrnap v0.8. The prophages was predicted using PHAge Search Tool. The gene island (GI) was predicted with Islander v1.2. The predicted CDSs were annotated from the NCBI non-redundant (NR) database, the databases of Swiss-Prot, The Pfam database, Cluster of Orthologous Groups of proteins (COGs), Gene Ontology (GO), Kyoto Encyclopedia of Genes and Genomes (KEGGs), Carbohydrate-Active enzymes (CAZy), Comprehensive Antibiotic Resistance Database (CARD) and Pathogen Host Interactions (PHI) using sequence alignment tools such as BLAST v2.3.0+, Diamond v0.8.35 and HMMER v3.1b2 [52].

### 4.7. Average Nucleotide Identity and Alignment Fraction

The average nucleotide identity (ANI) and alignment fraction (AF) were determined for 8 genomes as shown below using published methods [53,54]. ANI values were calculated for all pairwise comparisons. Custom scripts were used to perform these analyses and generate the ANI dendrogram. Pheatmap3 v 1.0.12 (R packages) was used to visualize the results.

The selected genome sequences were as follows: *Methylorubrum populi* BJ001, *Methylorubrum extorquens* CM4, *Methylobacterium salsuginis*, *Methylorubrum rhodinum*, *Methylobacterium organophilum*, *Methylobacterium brachiatum* TX0642, *Methylobacterium aquaticum* MA-22A.

### 4.8. Collinearity Analysis

The collinearity analysis was performed using BLASTN v2.80 (*E*-value cutoff of 10–15) using the *Methylorubrum populi* YC-XJ1 genome as the query against the *Methylorubrum populi* BJ001 genome [55]. The visualization was plotted by the SVG (v2.84 https://metacpan.org/release/SVG).

### 4.9. Sequence Analysis of qpeh2 and deph1

Nucleotides and amino acid sequence analysis of the *qpeh2* and *deph1* were performed using OMIGA 2.0. Blastn and Blastp tools were used for nucleotide and amino acid sequence identity searches, respectively. Phylogenetic tree analysis of the protein sequences was performed using MEGA 5.0 software by neighbor-joining method, Bootstrapping of 1000 replicates and Poisson correction. Homologous sequence alignment with the closest relative hydrolase was performed by ClustalW v2.1, and ESPript (v3.0, http://espript.ibcp.fr/ESPript/ESPript/). The presence of a signal peptide was predicted using signaIP v4.0.

### 4.10. Cloning, Expression and Purification of the Recombinant QPEH2 and DEPH1

The *qpeh2* and *deph1* genes were PCR-amplified from the genomic DNA of strain YC-XJ1 using the primers containing with *Bam*H I and *Hin*d III sites (Table S4). The PCR products were inserted into the expression vector pET-32a (+) to generate the recombinant plasmid pET-*qpeh2* and pET-*deph1*. The overexpression and purification followed the method described by [56].

### 4.11. Biochemical Properties of the Purified Recombinant QPEH2 and DEPH1

The degradation characteristics of QPEH2 for QPE and DEPH1 for DEP were detected. The optimal reaction pH was determined by incubating the reaction mixtures at 30 °C for 10 min in the following buffers: 10 mM Na_2_HPO_4_-Citric acid buffer, pH 3.0–7.0; 50 mM Tris-HCl buffer, pH 7.0–9.0; and 50 mM Glycine-NaOH buffer, pH 9.0–11.0. The optimal reaction temperature was assessed under pH 7.0 in 10 mM PBS buffer and incubating the reaction mixtures at different temperatures (15–50 °C) for 10 min. The residual concentration was assayed as described above 4.3. Each treatment was performed in three replicates, and the samples without enzyme solution was set as a control.

The effects of potential inhibitors or activators on the enzymatic activity were analyzed by the addition of various metal ion to the reaction mixture in 10 mM PBS buffer (pH 7.0), including Cd^2+^, Hg^2+^, Pb^2+^, Zn^2+^, Cu^2+^, Mn^2+^, Ni^2+^, Co^2+^, Mg^2+^, Fe^3+^, Fe^2+^, Ca^2+^ (1.0 mM). The reaction mixtures were preincubated for 10 min at 30 °C with each inhibitor or activator and the residual concentration was assayed as described above 4.3. Each treatment was performed in three replicates, and the samples without any additive was set as a control.

Enzymatic activities towards other substrates were performed in 10 mM PBS buffer (pH 7.0) for 30 min at 30 °C. The residual substrate concentration was assayed as described above 4.3. Each treatment was performed in three replicates, and the samples without corresponding enzyme was set as a control.

Metabolites of QPE degradation by QPEH2 were identified by HPLC-MS using the method described as [57].

### 4.12. Determination of Kinetic Constants

For kinetic studies, the purified recombinant protein was quantified with the BCA protein assay kit (TIANGEN Biotech Co., Ltd., Beijing, China), and one unit of enzymatic activity was defined as the amount of enzyme required to hydrolyze 1 µmol of substrate per minute. 120 µg of purified QPEH2 was assayed at varying QPE concentrations (0.2–2.4 mM) under conditions (25 °C, pH 7.0) in 10 mM PBS buffer. 500 µg of purified DEPH1 was assayed at varying DEP concentrations (0.2–2.4 mM) under conditions (25 °C, pH 7.0) in 10 mM PBS buffer. Kinetic parameters (*K*_m_ and *V*_max_) were calculated using a Lineweaver-Burk plot. The specificity constant, *K*_cat_/*K*_m_, was calculated to determine the substrate specificity. No more than 10% of the substrate was hydrolyzed during the assay. Each experiment was carried out in triplicate and samples without enzyme solution were used as a control.

### 4.13. Accession Numbers

The genomic sequences of *Methylobacterium populi* YC-XJ1 and the nucleotide sequences of *qpeh1* gene and *qpeh1* gene have been deposited in the GenBank database under accession numbers CP039546, submission ID 2345750 and 2345671, respectively. The strain YC-XJ1 has been deposited in China General Microbiological Culture Collection Center (CGMCC) under accession number CGMCC 18350.

### 4.14. Statistical Analysis

All the statistical analysis was performed by SPSS 13.0 as described in [7].

## 5. Conclusions

The strain YC-XJ1 showed great degrading ability towards a wide range of substrate, including AOPPs, OPFRs, PAEs, chlorpyrifos and phoxim. Genome annotation information showed that the strain YC-XJ1 contained a large number of exogenous compounds degradation pathways and hydrolase resources. QPEH2 and DEPH1 were proved to be members of the esterase family V and VII with hydrolysis activity to QPE and DEP. It was hoped that the *Methylobacterium populi* YC-XJ1 could be modified as engineered bacteria by transgenic engineering technology for practical application in bioremediation of polluted sites.

## Figures and Tables

**Table 1:** Details of genes in type of “xenobiotics biodegradation and metabolism”.

Pathway ID	Description	Gene No.
ko00982	Drug metabolism—cytochrome P450	13
ko00980	Metabolism of xenobiotics by cytochrome P450	12
ko00791	Atrazine degradation	10
ko00362	Benzoate degradation	8
ko00930	Caprolactam degradation	8
ko00627	Aminobenzoate degradation	8
ko00625	Chloroalkane and chloroalkene degradation	7
ko00643	Styrene degradation	7
ko00983	Drug metabolism—other enzymes	7
ko00361	Chlorocyclohexane and chlorobenzene degradation	4
ko00633	Nitrotoluene degradation	3
ko00621	Dioxin degradation	2
ko00626	Naphthalene degradation	2
ko00622	Xylene degradation	2
ko00364	Fluorobenzoate degradation	1
ko00624	Polycyclic aromatic hydrocarbon degradation	1
ko00623	Toluene degradation	1

## Supplementary Materials

The following are available online at https://www.mdpi.com/1422-0067/21/12/4436/s1, Table S1: Statistical table of gene function annotation of strain YC-XJ1, Table S2: The information of the protein sequences, Table S3: The detection method details of the substrate, Table S4: The amplification primer of *qpeh2* and deph1, Figure S1: The optimal pH of TPhP-degrading by strain YC-XJ1, Figure S2: Phylogenetic trees based on average nucleotide identity (ANI), Figure S3: Collinearity analysis of YC-XJ1 genome and BJ001 genome, Figure S4: Annotation statistics of COG categories of YC-XJ1 genome, Figure S5: Annotation statistics of GO categories of YC-XJ1 genome, Figure S6: Annotation statistics of CAZy database of YC-XJ1 genome, Figure S7: The summary of metabolic pathways of strain YC-XJ1 in KEGG database, Figure S8: Pathway classification of YC-XJ1 genome annotated by KEGG, Figure S9: The sequence alignment of DEPH1 with the most closely related proteins., Figure S10: The phylogenetic analysis of DEPH1, Figure S11: SDS-PAGE analysis of DEPH1 purification, Figure S12: The standard curve of substrate.
