# Supplementary material for: The Genome Analysis of Methylobacterium populi YC-XJ1 with Diverse Xenobiotics Biodegrading Capacity and Degradation Characteristics of Related Hydrolase
Source: Int J Mol Sci. 2020 Jun 22;21(12):4436. doi: 10.3390/ijms21124436 (PMC7352507; doi:10.3390/ijms21124436)
Supplement: Supplementary file 1 [file ijms-21-04436-s001.zip › Supplementary for conversion/Supplementary for conversion.pdf]

Supplementary

Table S1. Statistical table of gene function annotation of strain YC-XJ1.

Database	Number	Percentage
COG	4170	76.61%
GO	3458	64.33%
KEGG	2310	42.98%
Swiss-Prot	3174	58.31%
Nr	3547	65.17%
Pfam	3853	70.79%
CAZy	166	3.09%
VFDB	435	8.0%
CARD	249	4.57%
PHI	659	12.11%
Total	5375	98.75%

Table S2. The information of the protein sequences.

Accession Number	Organism	Size(aa)
ANT80587.1	*Pseudomonas* sp. J-5	309
ADW65729.1	*Pseudomonas azotoformans*	332
AJF83912.1	*Acinetobacter baumannii*	308
AHZ32245.1	*Aquamicrobium* sp. FpB-1	265
AOO35454.1	*Ochrobactrum* sp.	382
ARD08700.1	*Rhodococcus sp. JT-3*	393
AGZ87951.1	*Rhodococcus sp. JPL-2*	379
AEK27381.1	*Rhodococcus sp. T1*	379
P40601	*Photorhabdus luminescens*	645
O52756	*Salmonella typhimurium*	480
Q59798	*Streptomyces albus*	304
P19833	*Moraxella* sp.	319
Q56008	*Streptomyces* sp.	310
O52270	P*seudomonas* sp.	308
P24484	*Moraxella* sp.	433
Q53547	*Pseudomonas fluorescens*	218
Q53415	*Arthrospira platensis*	207
P37967	*Bacillus subtilis*	489
Q01470	*Pseudarthrobacter oxydans*	493
Q9Z545	*Streptomyces coelicolor*	502
O87861	*Streptomyces anulatus*	389
Q44050	*Arthrobacter globiformis*	375
Q53403	*Pseudomonas fluorescens*	382
Q59260	*Geobacillus thermocatenulatus*	417
Q52614	*Proteus vulgaris*	290
Q05489	*Burkholderia glumae*	358
Q9Z4M7	*Staphylococcus epidermidis*	643
P10335	*Staphylococcus aureus*	690
P08658	*Pseudomonas fragi*	293
O68551	*Pseudomonas luteola*	360
O66015	*Geobacillus stearothermophilus*	417
P9541	*Pseudomonas aeruginosa*	311
P24640	*Moraxella* sp.	315
Q02104	*Psychrobacter immobilis*	317
Q57427	*Haemophilus influenzae*	287
AAC41424	*Cupriavidus necator*	364
AAB89533.1	*Archaeoglobus fulgidus DSM 4304*	311
AEW03609.1	*Sulfobacillus acidophilus DSM 1033*	304

AJO67804	*Sphingobium sp. SM42*	302
AUH67707.1	*Gordonia sp. YC-JH1*	355
P24484	*Lipase 2-Moraxella sp. TA144*	433
Q9US38	*Schizosaccharomyces pombe*	341
P9WK86	*Mycobacterium tuberculosis*	319
A0A169RBE1	*Methylorubrum populi*	346
C5B0J6	*Methylorubrum extorquens*	366
C7CGE7	*Methylorubrum extorquens*	347

Table S3. The detection method details of the substrate.

**Detection Substrate (Wavelength, Retention Time)**	**HPLC-Method-1**
Benzene (260 nm, 1.79 min)<br>Phenol (260 nm, 4.68 min)<br>Pyrocatechol (280 nm, 2.7 min)<br>Phthalic acid (210 nm, 3.25 min)<br>Benzoic acid (230 nm, 4.69 min)<br>Salicylic acid (230 nm, 3.32 min)	The HPLC system (Agilent 1200) equipped with column ZORBAX Eclipase XDB C18 (4.6 mm×150 mm×5 μm) and DAD (diode-array detector). The software Chemstation (version 2.3, Agilent) was used for the analysis of the results. The detection parameters were as follows:<br>Column temperature: 30 °C<br>Elution: 60% methanol + 40% deionized water<br>Flow rate: 1 mL/min<br>Injection volume: 3 μL
Detection substrate (wavelength, retention time)	HPLC-method-2
Quizalofop-p-ethyl (236 nm, 2.42 min)<br>Clodinafop-propargyl (230 nm, 2.35 min)<br>Chlorpyrifos (205 nm, 2.42 min)<br>TPP (205 nm, 2.13 min)<br>Phoxim (275 nm, 2.1 min)	The same word as above<br>Column temperature: 30 °C<br>Elution: 100% methanol<br>Flow rate: 0.8 mL/min<br>Injection volume: 5 μL
Detection substrate (wavelength, retention time)	HPLC-method-3
4-Chlorobenzoic acid (230 nm, 2.9 min)	The same word as above<br>Column temperature: 30 °C<br>Elution: 70% methanol + 30% phosphoric acid solution (0.05%)<br>Flow rate: 1.0 mL/min<br>Injection volume: 3 μL
Detection substrate (wavelength, retention time)	HPLC-method-4
QPE (236 nm, 2.9 min)<br>QP (236 nm, 1.4 min)<br>CYP (236 nm, 2.4 min)	The same word as above<br>Column temperature: 30 °C<br>Elution: 95% methanol + 5% deionized water<br>Flow rate: 0.8 mL/min<br>Injection volume: 5 μL
Detection substrate (retention time)	GC-method-5

TCEP (2.203 min)<br>TDCPP (3.678 min)<br>DPP (3.663 min)<br>DBP (2.528 min)<br>DEP (2.528 min)	GC system (GC-2010 SHIMADZU, Japan) equipped with a RTX-1301 capillary column (30.0 m× 0.25 mm× 0.25 μm) and FID (flame ionization detector). The detection parameters were as follows: inlet temperature was 300 °C; column temperature was 280 °C; detector temperature was 300 °C; nitrogen (purity > 99,999%) was used as the carrier gas (30 mL per minute); and the injection volume was 1.0 μL. The GC solution software (version 2.32.00, SHIMADZU) was used for the analysis of the GC results.

Table S4. The amplification primer of *qpeh2* and *deph1*.

Name of Primer	Sequence
qpeh2-F	GCGC***GAATTC***ATGTTGCGGATGTTAAGC
qpeh2-R	GCGC***AAGCTT***CTTCACGGTCGGGAAG
deph1-F	GCGC***GAATTC***ATGACGCCCGCCGAG
deph1-R	GCGC***AAGCTT***TCAGCAGGCGGCGGCCA

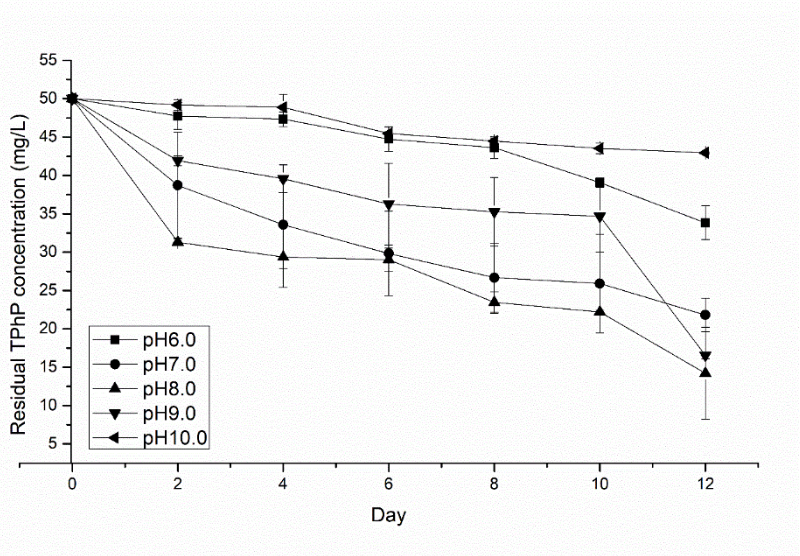

Figure S1. The optimal pH of TPhP-degrading by strain YC-XJ1.

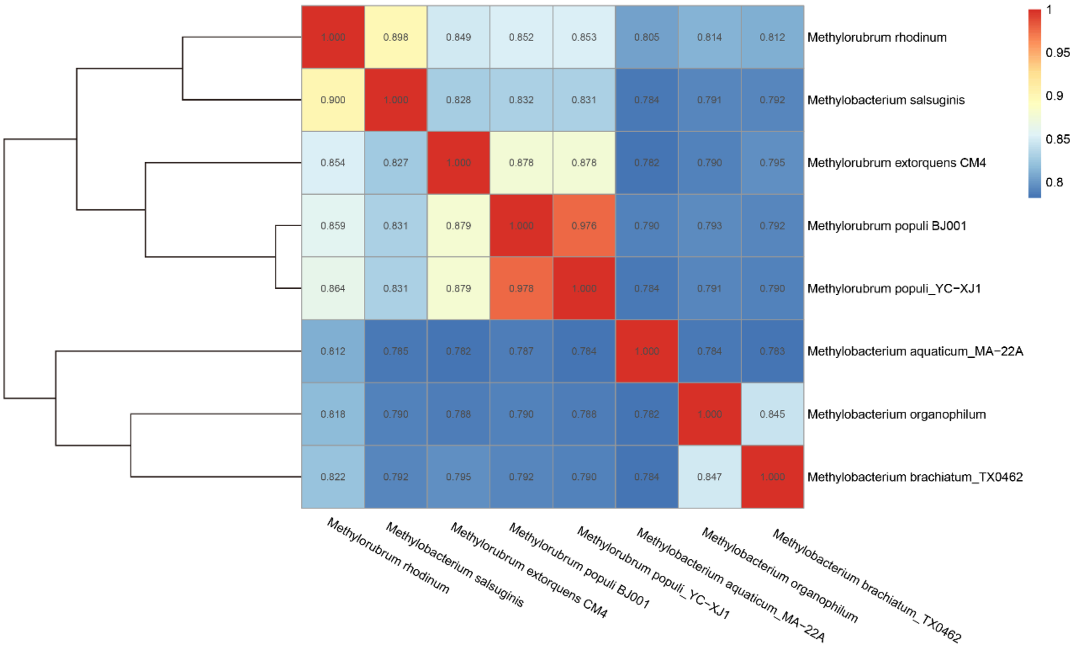

Figure S2. Phylogenetic trees based on average nucleotide identity (ANI).

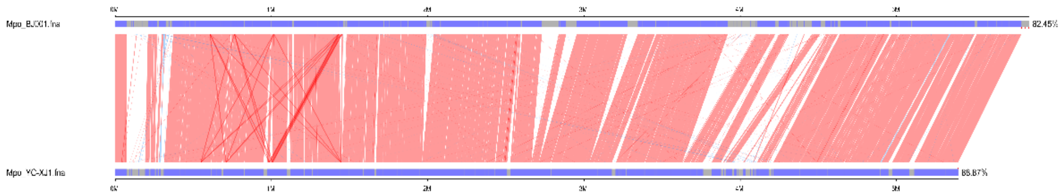

Figure S3. Collinearity analysis of YC-XJ1 genome and BJ001 genome.

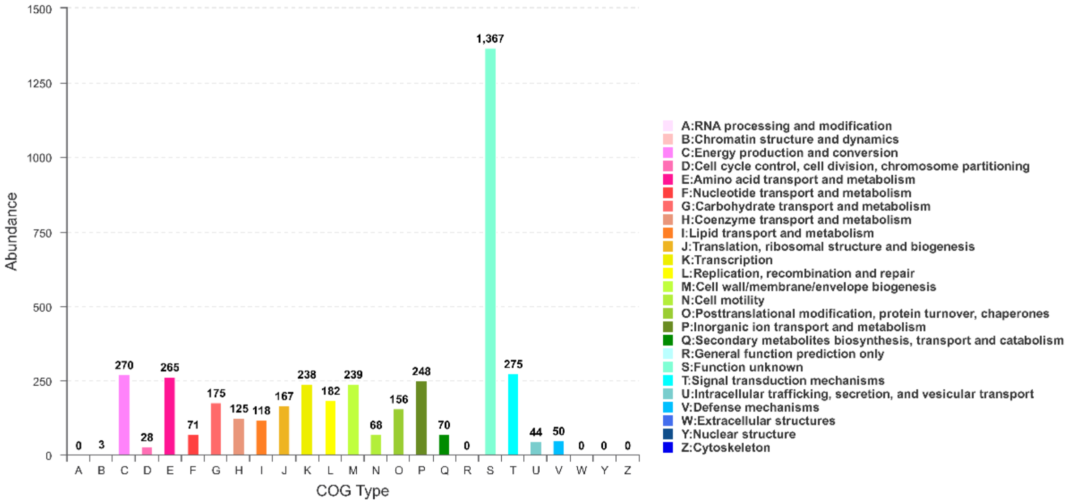

Figure S4. Annotation statistics of COG categories of YC-XJ1 genome.

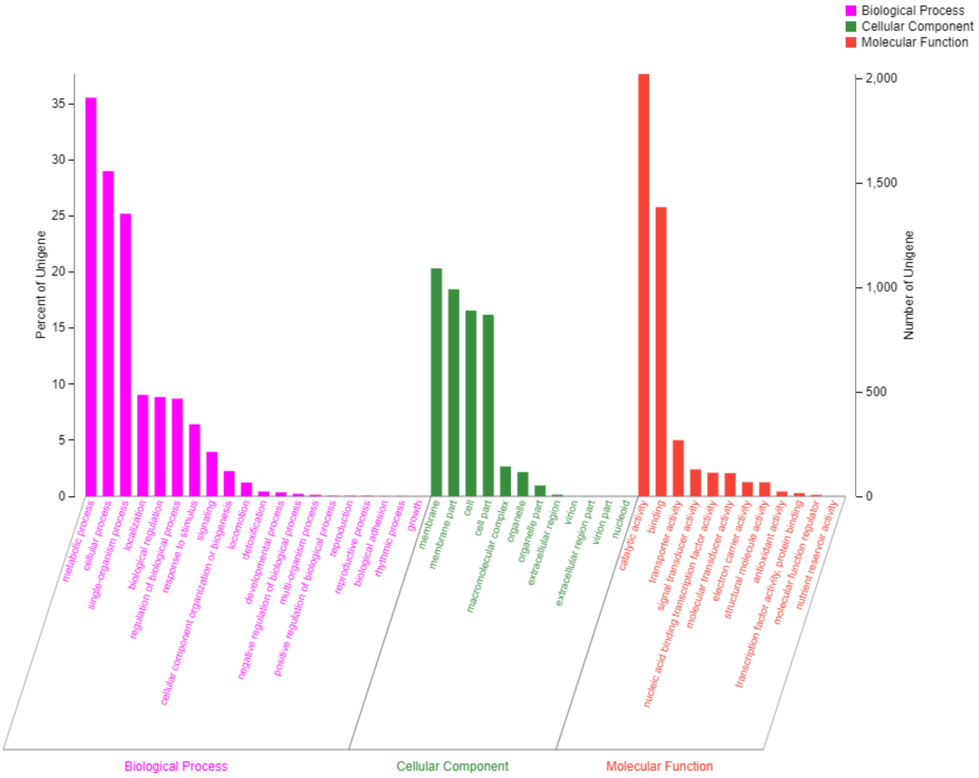

Figure S5. Annotation statistics of GO categories of YC-XJ1 genome.

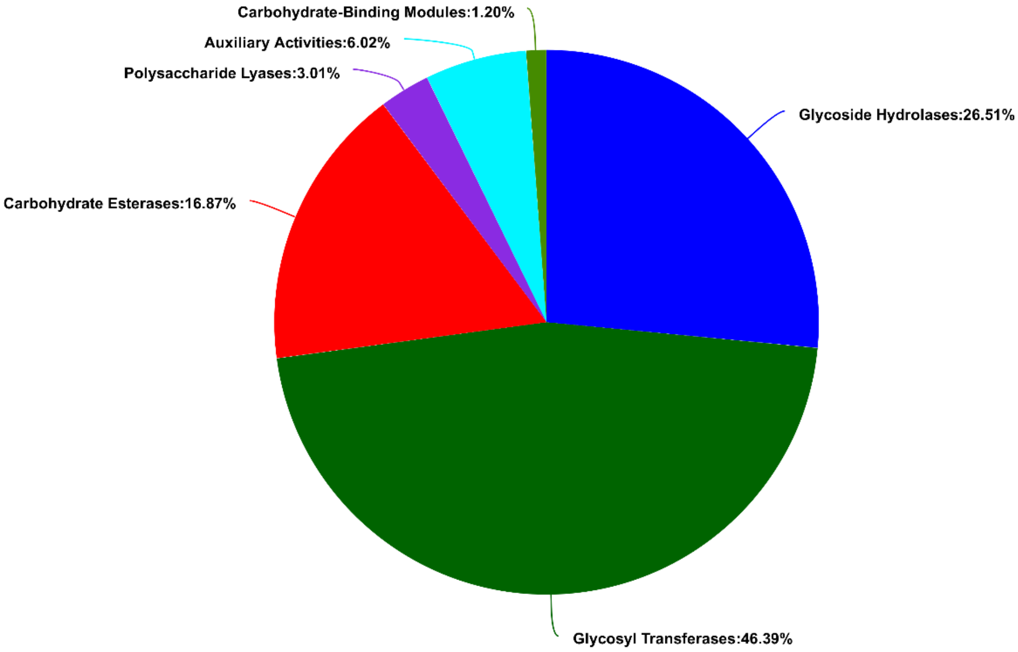

Figure S6. Annotation statistics of CAZy database of YC-XJ1 genome.

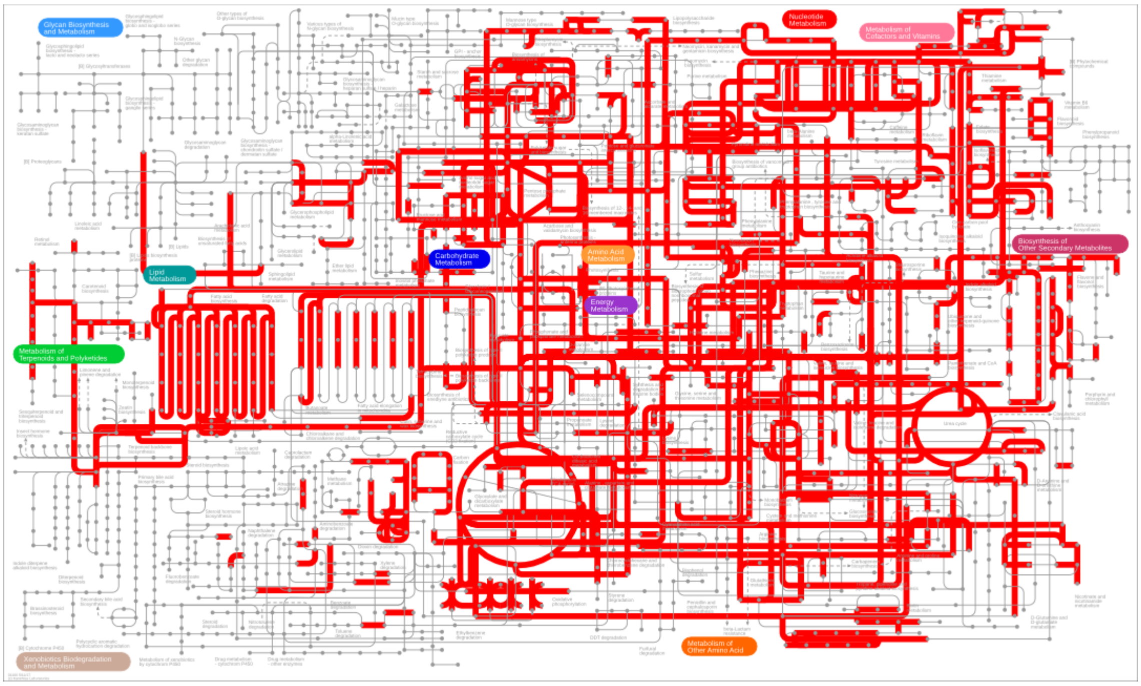

Figure S7. The summary of metabolic pathways of strain YC-XJ1 in KEGG database. The red dots represented the annotated pathways, the grey dots represented the unannotated pathways.

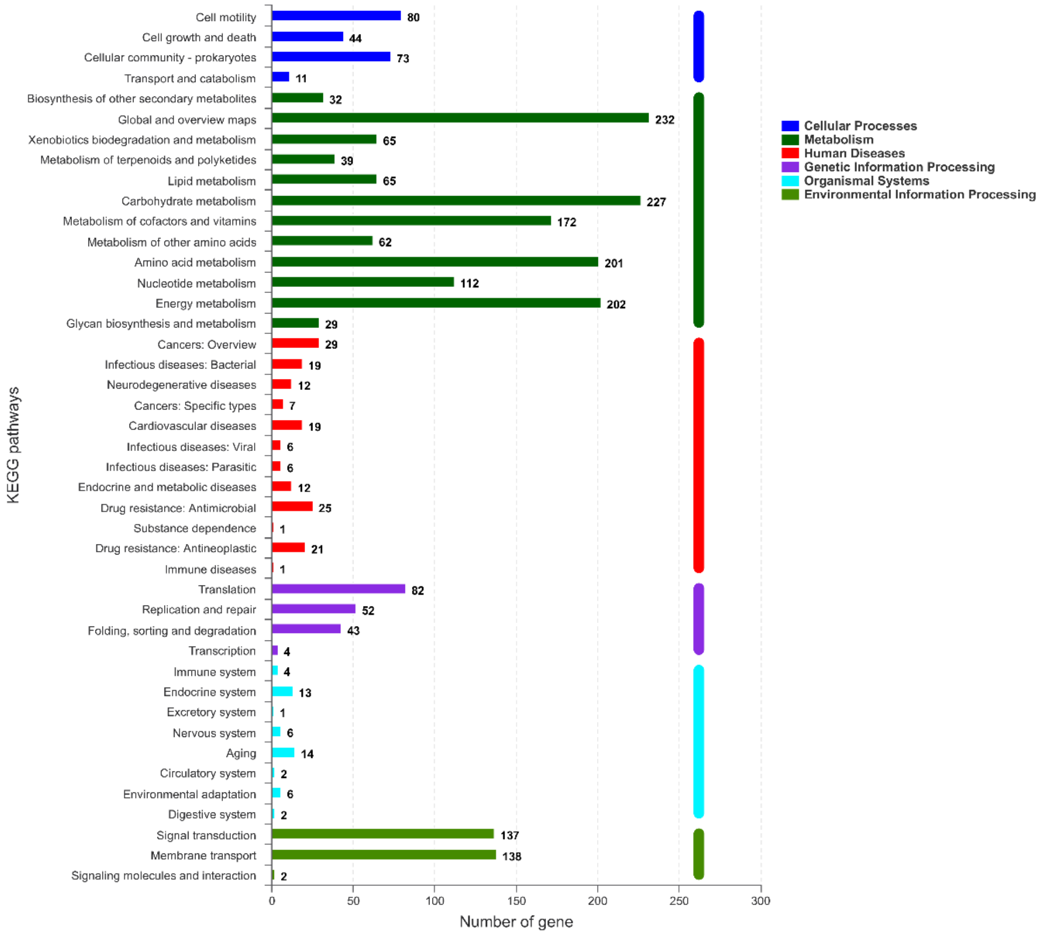

Figure S8. Pathway classification of YC-XJ1 genome annotated by KEGG.

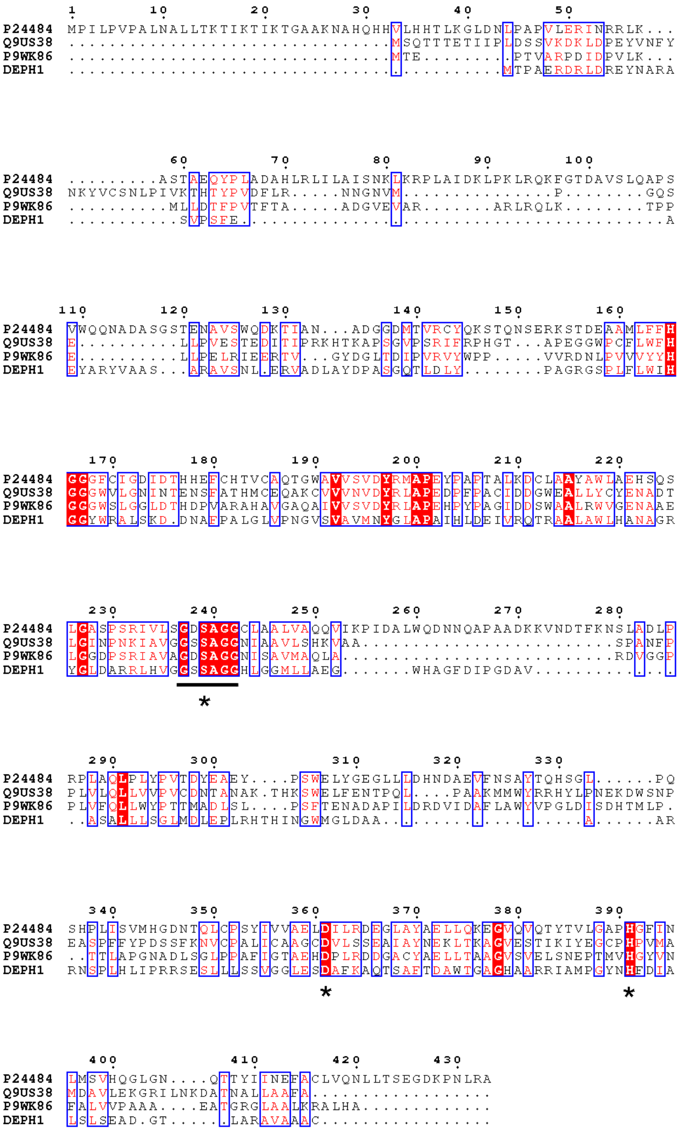

Figure S9. The sequence alignment of DEPH1 with the most closely related proteins. P24484, Lipase 2 of *Moraxella* sp. TA144, available in UniProt Knowledgebase; Q9US38, α/β hydrolase superfamily protein of *Schizosaccharomyces pombe* 972/ATCC 24843; P9WK86, Carboxylesterase NlhH of *Mycobacterium tuberculosis* CDC 1551/Oshkosh, available in the UniProt Knowledgebase. The conserved hydrolase motif (G-X-S-X-G) was underlined, and the amino acids that form the catalytic triad (Ser-Asp-His) was indicated by asterisks. The identical amino acid residues were shown in red color.

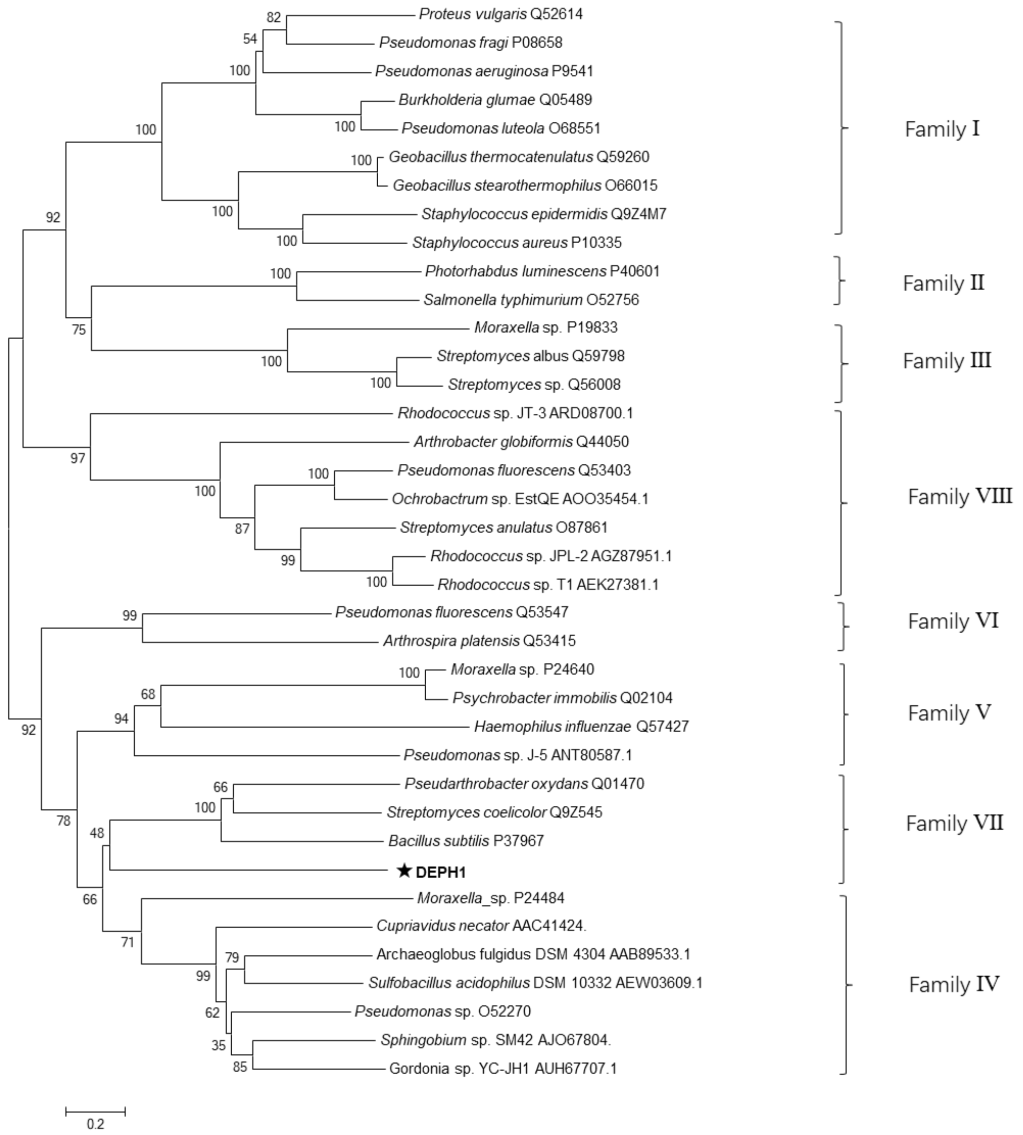

Figure S10. The phylogenetic analysis of DEPH1. The phylogenetic tree was constructed using Mega 5.0 by the neighbor-joining method, bootstrapping of 1000 replicates and Poisson model, and the details of sequences were showed in Table S2.

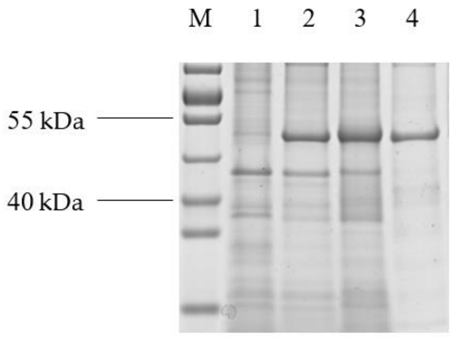

Figure S11. SDS-PAGE analysis of DEPH1 purification. Lane M: protein marker; lane 1: total protein of *E. coli* (pET32a); lane 2: total protein of E. coli (pET32a-*deph1*) after induction with IPTG; lane 3: supernatant of *E.coli* (pET32a-*deph1*) after induction with IPTG; lane 4: purified recombinant DEPH1.
